# Supplementary material for: Riemannian Geometry of Functional Connectivity Matrices for Multi-Site Attention-Deficit/Hyperactivity Disorder Data Harmonization
Source: Front Neuroinform. 2022 May 23;16:769274. doi: 10.3389/fninf.2022.769274 (PMC9171428; doi:10.3389/fninf.2022.769274)
Supplement: Supplementary file 1 [file Data_Sheet_1.zip › Data sheet 1.pdf]

Appendix

EQUIVALENCE BETWEEN PARALLEL TRANSPORT AND MATRIX WHITENING

In general, matrix whitening and parallel transport using $\overline{\Sigma}$ as the reference point are different transformations. However, we show here that under the condition $\left[\overline{\Sigma}, \overline{\Sigma}^{(k)}\right] = 0, \forall k$, both frameworks are equivalent. To this aim, consider the following property regarding the unique positive-definite square root of the product of two $n \times n$ SPD matrices:

Property: Let A and B be two $n \times n$ SPD matrices that commute, i.e., $[A, B] \equiv AB - BA = 0$. Then $(AB)^{\frac{1}{2}} = A^{\frac{1}{2}} B^{\frac{1}{2}}$.

Proof: Considering that the unique SPD square root of some SPD matrix X can be computed as $X^{\frac{1}{2}} = \mathrm{Exp}(\frac{1}{2}\mathrm{Log}(X))$, and taking into account that, given two SPD matrices A and B such that $[A, B] = 0$, $\mathrm{Exp}(A + B) = \mathrm{Exp}(A)\,\mathrm{Exp}(B)$ and $\mathrm{Log}(AB) = \mathrm{Log}(A) + \mathrm{Log}(B)$:

$$\begin{aligned}(AB)^{\frac{1}{2}} &= \mathrm{Exp}(\frac{1}{2}\mathrm{Log}(AB)) = \mathrm{Exp}\big(\frac{1}{2}(\mathrm{Log}(A) + \mathrm{Log}(B))\big) = \\ &= \mathrm{Exp}(\frac{1}{2}\mathrm{Log}(A))\,\mathrm{Exp}(\frac{1}{2}\mathrm{Log}(B)) = A^{\frac{1}{2}} B^{\frac{1}{2}} \quad \square\end{aligned} \tag{S1}$$

Now, recall that the overall parallel transport transformation (including the last matrix whitening step) when using as the reference point Σ_0 the global mean is (subsection 2.4.2):

$${\Sigma_i}^{(k)} \to \overline{\Sigma}^{-1/2} \left(\overline{\Sigma}\,(\overline{\Sigma}^{(k)})^{-1}\right)^{1/2} {\Sigma_i}^{(k)} \left(\left(\overline{\Sigma}\,(\overline{\Sigma}^{(k)})^{-1}\right)^{1/2}\right)^T \overline{\Sigma}^{-1/2}. \tag{S2}$$

Supposing that $\overline{\Sigma}$ and $(\overline{\Sigma}^{(k)})^{-1}$ commute, by virtue of the previously stated property and taking into account that given two matrices A and B, $(AB)^T = B^T A^T$:

$$\begin{aligned}{\Sigma_i}^{(k)} &\to \overline{\Sigma}^{-1/2} \left(\overline{\Sigma}\,(\overline{\Sigma}^{(k)})^{-1}\right)^{1/2} {\Sigma_i}^{(k)} \left(\left(\overline{\Sigma}\,(\overline{\Sigma}^{(k)})^{-1}\right)^{1/2}\right)^T \overline{\Sigma}^{-1/2} = \\ &= \overline{\Sigma}^{-1/2}\,\overline{\Sigma}^{1/2} \left((\overline{\Sigma}^{(k)})^{-1}\right)^{1/2} {\Sigma_i}^{(k)} \left(\left((\overline{\Sigma}^{(k)})^{-1}\right)^{1/2}\right)^T \left(\overline{\Sigma}^{1/2}\right)^T \overline{\Sigma}^{-1/2} = \\ &= \overline{\Sigma}^{-1/2}\,\overline{\Sigma}^{1/2}\,(\overline{\Sigma}^{(k)})^{-1/2} {\Sigma_i}^{(k)}\,(\overline{\Sigma}^{(k)})^{-1/2}\,\overline{\Sigma}^{1/2}\,\overline{\Sigma}^{-1/2} = \\ &= (\overline{\Sigma}^{(k)})^{-1/2} {\Sigma_i}^{(k)}\,(\overline{\Sigma}^{(k)})^{-1/2},\end{aligned} \tag{S3}$$

where in the second step we have used the facts that for a SPD matrix A, we take $A^{1/2}$ to be also SPD and thus satisfies $\left(A^{1/2}\right)^T = A^{1/2}$, along with $\left((A)^{-1}\right)^{1/2} = (A)^{-1/2}$, with $(A)^{-1/2}$ also SPD, and then, $\left((A)^{-1/2}\right)^T = (A)^{-1/2}$. One can conclude that under vanishing commutators $\left[\overline{\Sigma}, (\overline{\Sigma}^{(k)})^{-1}\right]$, the transformation reduces to matrix whitening. Since $\overline{\Sigma}$ and $(\overline{\Sigma}^{(k)})^{-1}$ belong to $GL(n)$, the requirement

$\left[\overline{\Sigma}, (\overline{\Sigma}^{(k)})^{-1}\right] = 0$ is equivalent to $\left[\overline{\Sigma}, \overline{\Sigma}^{(k)}\right] = 0$. Therefore, when site means $\overline{\Sigma}^{(k)}$ commute with the global mean $\overline{\Sigma}$, parallel transport and matrix whitening represent equivalent frameworks.

PROPERTIES OF RIGID LOG-EUCLIDEAN TRANSLATION

As mentioned in the description of Rigid Log-Euclidean Translation in subsection 2.4.3, the transformation preserves intra-site geodesic distances under the LERM framework and displaces the matrices in such a way that their transformed site mean $\tilde{\overline{\Sigma}}^{(k)}$ is the global mean $\overline{\Sigma}$, for all sites k.

By using the definition of LERM geodesic distance (7) for matrices belonging to the same site k and modified according to RLET($\overline{\Sigma}$) transformation (14) one finds

$$\begin{aligned} & d(\tilde{\Sigma}_i^{(k)}, \tilde{\Sigma}_j^{(k)}) = \\ & = ||\mathrm{Log}(\tilde{\Sigma}_i^{(k)}) - \mathrm{Log}(\tilde{\Sigma}_j^{(k)})||_F = \\ & = ||\mathrm{Log}(\overline{\Sigma}) + \mathrm{Log}(\Sigma_i^{(k)}) - \mathrm{Log}(\overline{\Sigma}^{(k)}) - \mathrm{Log}(\overline{\Sigma}) - \mathrm{Log}(\Sigma_j^{(k)}) + \mathrm{Log}(\overline{\Sigma}^{(k)})||_F = \\ & = ||\mathrm{Log}(\Sigma_i^{(k)}) - \mathrm{Log}(\Sigma_j^{(k)})||_F = d(\Sigma_i^{(k)}, \Sigma_j^{(k)}), \end{aligned} \tag{S4}$$

meaning that intra-site geodesic distances are preserved as expected.

It is also straightforward to prove that $\tilde{\overline{\Sigma}}^{(k)} = \overline{\Sigma}, \forall\, k$. Considering the definition of site mean (12) for matrices transformed according to (14):

$$\begin{aligned} \mathrm{Log}(\tilde{\overline{\Sigma}}^{(k)}) &= \frac{1}{N^{(k)}} \sum_{i \in (k)} \mathrm{Log}(\tilde{\Sigma}_i^{(k)}) = \\ &= \frac{1}{N^{(k)}} \left(\sum_{i \in (k)} \left(\mathrm{Log}(\overline{\Sigma}) + \mathrm{Log}(\Sigma_i^{(k)}) - \mathrm{Log}(\overline{\Sigma}^{(k)}) \right) \right) = \\ &= \frac{1}{N^{(k)}} \left(N^{(k)} \mathrm{Log}(\overline{\Sigma}) - N^{(k)} \mathrm{Log}(\overline{\Sigma}^{(k)}) \right) + \frac{1}{N^{(k)}} \sum_{i \in (k)} \mathrm{Log}(\Sigma_i^{(k)}) = \\ &= \mathrm{Log}(\overline{\Sigma}) - \mathrm{Log}(\overline{\Sigma}^{(k)}) + \mathrm{Log}(\overline{\Sigma}^{(k)}) = \mathrm{Log}(\overline{\Sigma}), \end{aligned} \tag{S5}$$

and therefore site means become the global mean $\overline{\Sigma}$. In the case where the term $\mathrm{Log}(\overline{\Sigma})$ is removed from the transformation rule (14), one gets $\mathrm{Log}(\tilde{\overline{\Sigma}}^{(k)}) = 0 = \mathrm{Log}(I)$, and site means become the identity matrix I.
